# Virulence and pathogenesis of SARS-CoV-2 infection in rhesus macaques: A nonhuman primate model of COVID-19 progression

**DOI:** 10.1371/journal.ppat.1008949

**Published:** 2020-11-12

**Authors:** Huiwen Zheng, Heng Li, Lei Guo, Yan Liang, Jing Li, Xi Wang, Yunguang Hu, Lichun Wang, Yun Liao, Fengmei Yang, Yanyan Li, Shengtao Fan, Dandan Li, Pingfang Cui, Qingling Wang, Haijing Shi, Yanli Chen, Zening Yang, Jinling Yang, Dong Shen, Wei Cun, Xiaofang Zhou, Xingqi Dong, Yunchuan Wang, Yong Chen, Qing Dai, Weihua Jin, Zhanlong He, Qihan Li, Longding Liu

**Affiliations:** 1 Institute of Medical Biology, Chinese Academy of Medical Sciences & Peking Union Medical College, Kunming, Yunnan Province, People’s Republic of China; 2 Yunnan Provincial Center for Disease Control and Prevention, Kunming, Yunnan Province, People’s Republic of China; 3 Yunnan Provincial Infectious Disease Hospital, Kunming, Yunnan Province, People’s Republic of China; Icahn School of Medicine at Mount Sinai, UNITED STATES

## Abstract

The COVID-19 has emerged as an epidemic, causing severe pneumonia with a high infection rate globally. To better understand the pathogenesis caused by SARS-CoV-2, we developed a rhesus macaque model to mimic natural infection via the nasal route, resulting in the SARS-CoV-2 virus shedding in the nose and stool up to 27 days. Importantly, we observed the pathological progression of marked interstitial pneumonia in the infected animals on 5–7 dpi, with virus dissemination widely occurring in the lower respiratory tract and lymph nodes, and viral RNA was consistently detected from 5 to 21 dpi. During the infection period, the kinetics response of T cells was revealed to contribute to COVID-19 progression. Our findings implied that the antiviral response of T cells was suppressed after 3 days post infection, which might be related to increases in the Treg cell population in PBMCs. Moreover, two waves of the enhanced production of cytokines (TGF-α, IL-4, IL-6, GM-CSF, IL-10, IL-15, IL-1β), chemokines (MCP-1/CCL2, IL-8/CXCL8, and MIP-1β/CCL4) were detected in lung tissue. Our data collected from this model suggested that T cell response and cytokine/chemokine changes in lung should be considered as evaluation parameters for COVID-19 treatment and vaccine development, besides of observation of virus shedding and pathological analysis.

## Author summary

Understanding of the pathologic process caused by SARS-CoV-2 is critical for promoting vaccine evaluations and medical treatment. Prior to the development of this model, several animal models of SARS-CoV-2 infection focused on revealing the virus shedding period, the development of interstitial pneumonia, and virus dissemination in respiratory tract. However, data describing the kinetics of the T cell response and local immune response during SARS-CoV-2 infection are lacking. Here, in our rhesus macaque model, in addition to focusing on virus shedding and interstitial pneumonia similar with human cases, we observed the response of T cell subsets and local cytokine/chemokine changes in respiratory tract regarded as the important evaluation parameters for a successful animal model of COVID-19.

## Introduction

On January 7, 2020, the Chinese health department confirmed that a new coronavirus was associated with the first cluster of cases of pneumonia in Wuhan, Hubei[1]. Since the genome of this new virus shares approximately 80% identity with that of severe acute respiratory syndrome coronavirus (SARS- CoV) [2], this new beta coronavirus was named as severe acute respiratory syndrome–coronavirus 2 (SARS-CoV-2), causing the newly described “coronavirus disease 2019” (COVID-19) in humans that is a rapidly spreading global outbreak. On January 30, 2020, the World Health Organization (WHO) announced the epidemic as a public health emergency of international concern. As of August 26, 2020, the COVID-19 has emerged as a severe epidemic, with more than 23,903,870 confirmed cases, of which 819,609 were fatal [3]. The latest data show that outside China, more than 215 countries have reported confirmed cases. The situation in the U.S., Brazil, and India is more serious than that in other countries [3].

Previous studies have reported that SARS-CoV and SARS-CoV-2 use the same receptor-angiotensin converting enzyme 2 (ACE2) for infection, mainly infect airway and alveolar epithelial cells, vascular endothelial cells and macrophages[4–7]. Similar to the lung pathology of severe acute respiratory syndrome (SARS), the lungs of patients with COVID-19 also exhibit pulmonary alveolar edema with hemorrhage, necrotizing bronchiolitis, alveolitis with inflammatory injury of epithelial cells, and other lung damage, accompanied by increased levels of IL-2, IL-7, IL-10, G-CSF, IP-10, MCP-1, MIP-1a and TNF-α, suggesting that there may be a cytokine storm related to the severity of the disease[8].

SARS-CoV-2 has a variety of transmission routes including respiratory droplets and close contact [9, 10], while the median time from symptom onset to diagnosis is 7 (4–8) days, and the estimated median incubation period is 4 (2–7) days[8, 11]. From the age distribution of patients worldwide, people of all ages are not resistant to SARS-CoV-2[12–14]. The close contacts of COVID-19 patients and latently infected people are the high-risk population of SARS-CoV-2 attacks[15].

Understanding the viral infection process is the first step of all effective means to control the disease and develop vaccines. In this case, establishing an effective animal model of SARS-CoV-2 infection can not only help us understand the pathological characteristics of COVID-19, but also help to clarify the viral systematic pathologic process induced by infection. Although human ACE2 transgenic mice infected with SARS-CoV-2 have been reported to study the pathogenicity of the virus[5, 16], the evaluation of vaccines in this model is limited because of the limited animal body size and the nonsystematic characteristics of the infection process. Previous reports have shown that rhesus macaques inoculated with SARS-CoV or Middle East respiratory syndrome (MERS-CoV) developed respiratory disease[17, 18]. However, whether rhesus macaque can be used as an animal model for studying SARS-CoV-2 infection remains to be further studied. Several studies have evaluated the rhesus macaque model[19–23], mainly focusing on the analysis of SARS-CoV-2 viral distribution and pathological changes but there is a lack of data on the systematic immunologic process induced by infection. Here, we present a rhesus macaque model nasally inoculated with SARS-CoV-2. In addition to observing viral replication in vivo and histopathological changes in interstitial pneumonia, we provide a more detailed evaluation of the systemic immune response in SARS-CoV-2-infected rhesus macaques, including immunological cell and inflammatory cytokine dynamic characteristics. In addition, we also compared our data with those from previous studies[19–23] to summarize the evaluation indexes of rhesus macaque models in this article.

## Results

### Clinical signs related to SARS-CoV-2 infection in rhesus macaques

**Fig 1:**
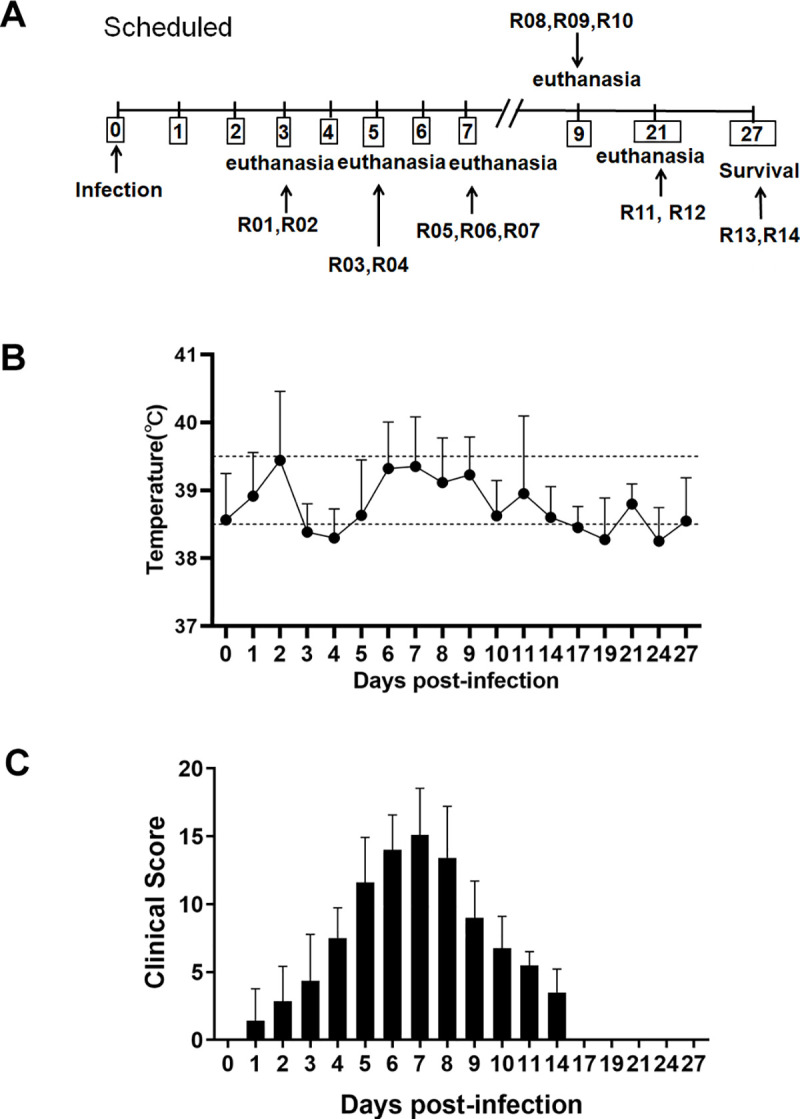
Experimental schedule and clinical signs in rhesus macaques inoculated with SARS-CoV-2. (A) Schedule of viral inoculation and necropsies. (B) Monitoring of the body temperature of infected animals for 27 days. (C) Animals were scored for 27 days, and their mean clinical score ±SD was calculated.

In total, 14 rhesus macaques were inoculated with SARS-CoV-2-KMS1/2020 via the intranasal route, and animals were assigned for scheduled necropsies at 3 (animal ID:R01, R02), 5 (animal ID:R03, R04), 7 (animal ID:R05, R06, R07), 9 (animal ID:R08, R09, R10), 21 (animal ID:R11, R12) days post infection(dpi); 2 additional animals (animal ID:R13, R14) were monitored for survival (Fig 1A). Clinical observation of SARS-CoV-2 infection in rhesus macaques showed a temporary rise in temperature on average 1–2 dpi, with peaking at 41.5°C in one monkey, but subsequently decreasing to 38.3°C on 3–4 dpi. Starting on 5 dpi, some animals showed another increase in body temperature beyond 39°C for 4 days, returning to a normal range on 14 dpi (Fig 1B). On 3–9 dpi, all animals showed less activity and loss of appetite. Clinical scores were evaluated based on an established scoring criterion for viral infection in rhesus macaques (S1 Table)[18]. We observed higher clinical scores between 4 and 10 dpi, and clinical scores returned to baseline by 11 dpi in the remaining animals (Fig 1C). There were no remarkable changes in clinical symptoms including cough, vomiting, and the absence of diarrhea.

### Long period of viral shedding following inoculation with SARS-CoV-2

**Fig 2:**
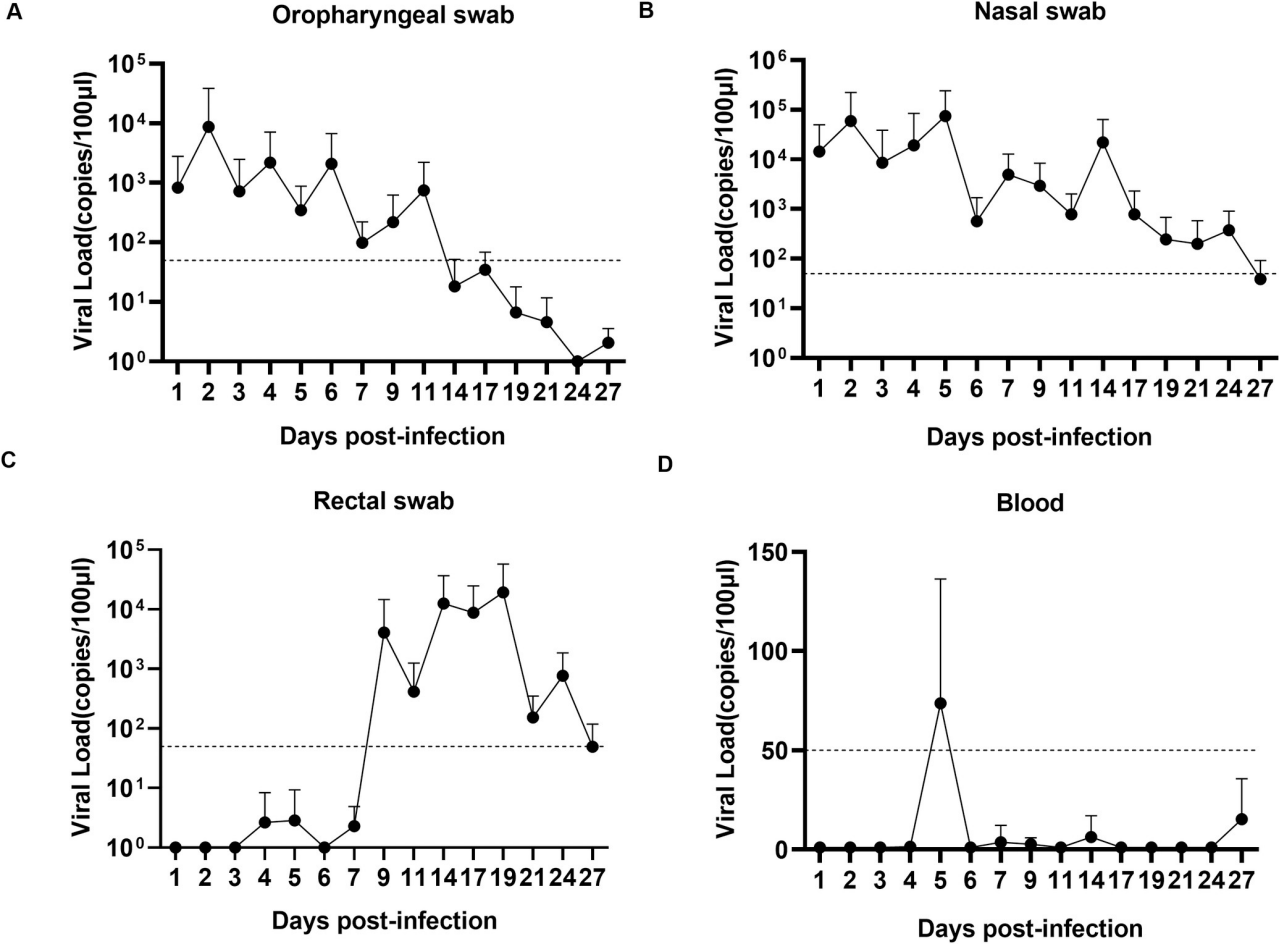
Viral shedding and viremia in rhesus macaques inoculated with SARS-CoV-2. Oropharyngeal (A), nasal (B), and rectal (C) swab samples and blood samples (D) were collected for 27 days. Of note, 2 animals were euthanized on 3, 5 and 21 dpi, and 3 animals were euthanized on 7 and 9 dpi; thus, samples were reduced in the plots. RNA was extracted, and the viral load was determined as copies per 100 μl. The dashed line represents the detected threshold.

The oropharyngeal, nasal and rectal swab samples were obtained daily from the animals after SARS-CoV-2 infection. From 2–14 dpi, SARS-CoV-2 RNA was detected by qRT-PCR in oropharyngeal swab samples from the infected animals, and viral RNA peaked to over 10^3^ copies on 2–6 dpi and was undetected on 17dpi (Fig 2A). Two peaks beyond 10^4^ copies of viral RNA load were observed in nasal swab samples from most rhesus macaques on 2 and 5 dpi. The viral RNA of the second peak in nasal swab samples peaked at approximately 10^5^ copies on 5 dpi. Notably, the SARS-CoV-2 viral shedding period in nasal tissue was much longer than that in oropharyngeal tissue, which lasted for 27 days in some animals (Fig 2B). Compared with nasal and oropharyngeal samples, the rectal swab samples were positive from 9–27 dpi (Fig 2C). Whereas clinical observations showed normal results in monkeys from 10 dpi, the above results indicated a long shedding time of SARS-CoV-2 in some animals without obvious clinical signs. Viremia was also detected in blood samples, with transiently positive results at 5 dpi (Fig 2D). To verify viral replication in vivo, we detected the infectious viral particles in rectal swab samples by a median tissue culture infectious dose (TCID_50_) assay. As a result, the infectious virus was found only in rectal samples on 9 dpi (S2 Table). Furthermore, we used viral RNA extracted from nasal swab samples on 3, 9 and 14 dpi, and viral spike (S), envelope protein (E), and nucleocapsid (N) genes showed 100% identity, indicating SARS-CoV-2 infection and replication in the animals (S1 Fig)

### Wide distribution of virus through the respiratory tract to other tissues

**Fig 3:**
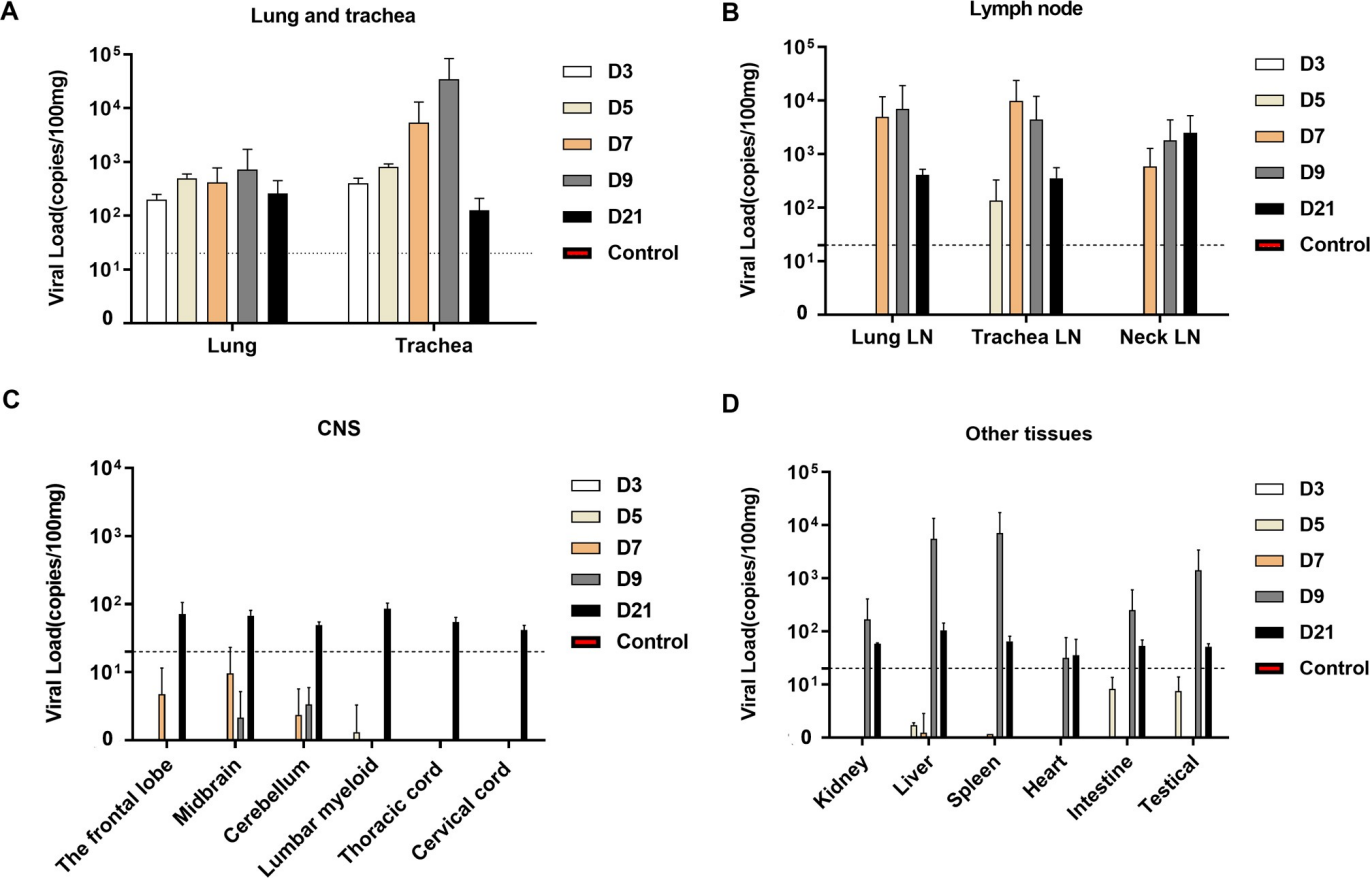
Viral load in tissues of rhesus macaques inoculated with SARS-CoV-2. Necropsy tissue samples of rhesus macaques were collected on 3, 5, 7, 9 and 21 dpi. RNA was extracted, and viral load was detected by qRT-PCR as copies per 100 mg tissue in (A) lungs and tracheas; (B) LNs; (C) CNS; and (D) other tissues. Control: the archived normal tissues. Error bars represent the SD.

To monitor for dissemination of virus in rhesus macaques during the infection period, tissues from necropsies were detected for viral RNA on 3, 5, 7, 9 and 21 dpi. The presence of viral RNA throughout the lungs and tracheas of inoculated rhesus macaques were observed from 3 to 9 dpi, with 10^2^-10^4^copies/100mg tissue of viral load peaking on 7–9 dpi (Fig 3A). Although the presence of viral RNA was displayed some variations between the different animals, we found that lymph nodes (LNs) may play a critical role in the dissemination of SARS-CoV-2 in vivo, as there were c higher viral loads(up to 10^4^copies/100mg tissue) that were consistently detected in the LNs from 5–21 dpi (Fig 3B). In addition to the respiratory tract and LNs, we analyzed the central nervous system (CNS) and other vital organ tissues. Viral RNA was detected in brain and spinal cords on 21 dpi (Fig 3C) and in kidney, liver, spleen, heart, intestine and testicle tissue samples, with higher viral loads in the late stage of infection on 9 dpi (Fig 3D), suggesting that nasal infection with SARS-CoV-2 can induce wide viral dissemination in rhesus macaques.

### Progressive pneumonia with extensive pathology of SARS-CoV-2 in the respiratory tract

**Fig 4:**
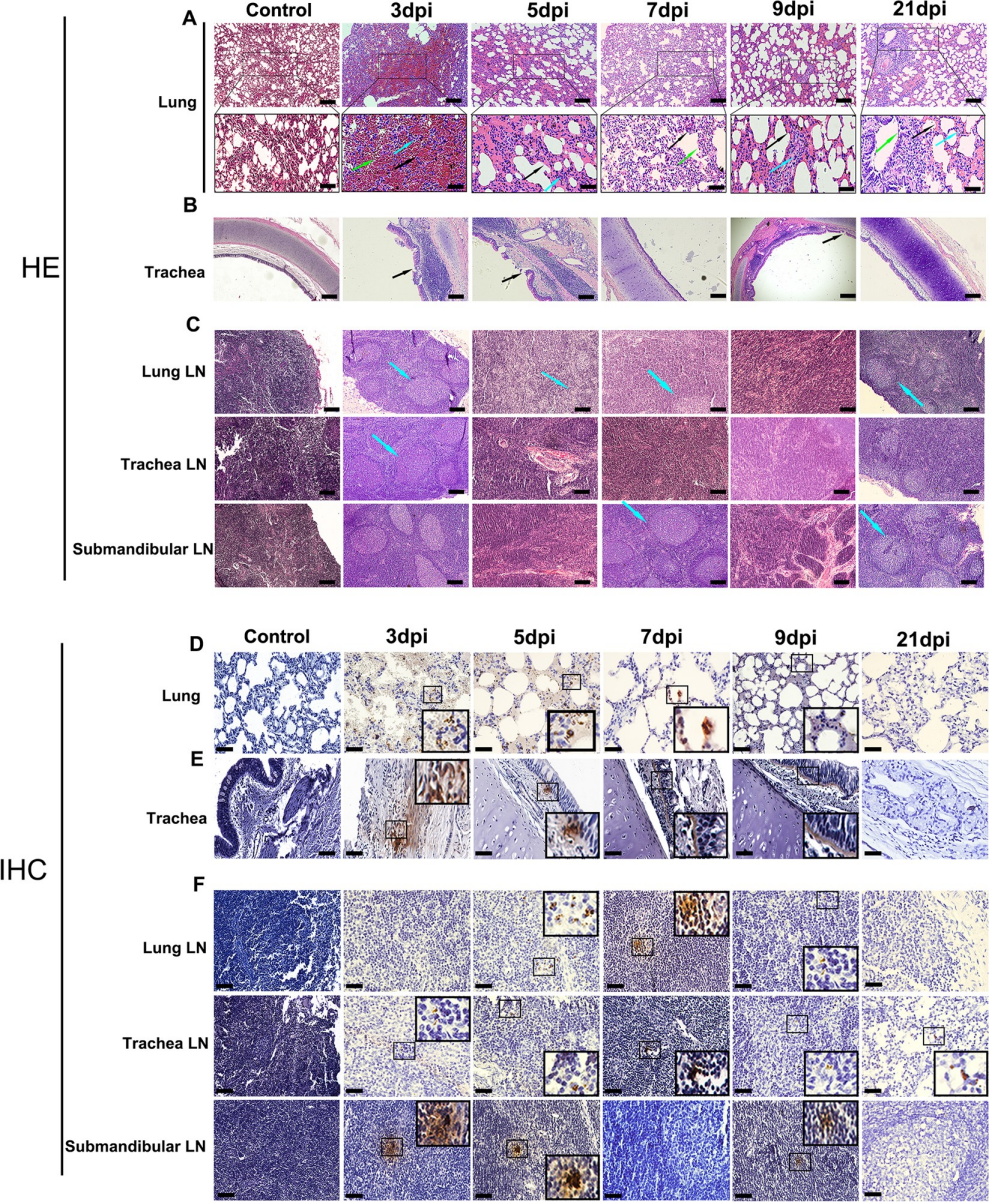
Histopathological changes in rhesus macaques inoculated with SARS-CoV-2. Necropsy tissue samples of rhesus macaques were collected on 3, 5, 7, 9 and 21 dpi, stained with H&E for histopathological evaluation, or labeled with anti-N antigen antibody for IHC analysis. (A) Lung tissue samples showed mild to marked acute interstitial pneumonia demonstrated by diffuse alveolar damage and edema (**black arrow**), alveolar thickening (green arrow), large numbers of neutrophils, macrophages, and infiltration (blue arrow). Anti-SARS-CoV-2 antigen for IHC revealed viral N antigen in alveolar pneumocytes. (B) Trachea tissues showed damage to epithelial cells (black arrow). (C) LN samples showed enlarged germinal centers (blue arrow) in response to viral infection. IHC reveals viral N antigen within LNs. (D) Lung (E) trachea and (F) LN IHC analyses (magnification: 400×). Yellow scale bar, 100μl; Black scale bar, 50μl. Control: the archived normal tissues.

**Fig 5:**
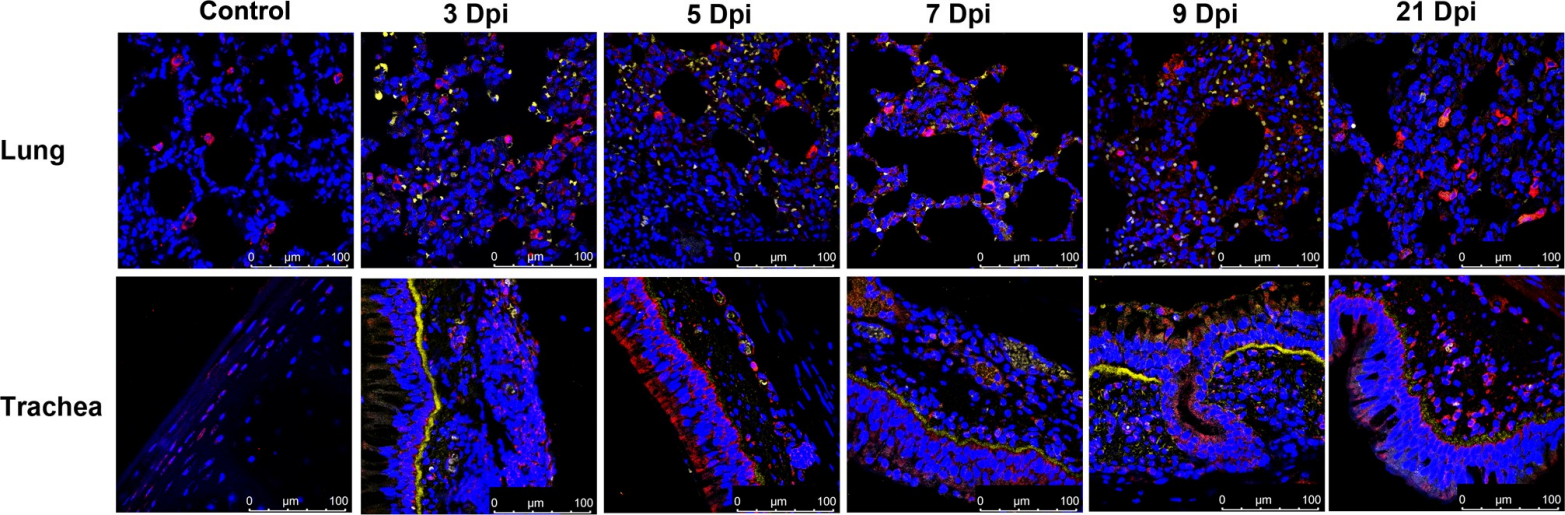
Analysis of ACE2 related to SARS-CoV-2 infection in rhesus macaques by confocal immunofluorescence assay. Human ACE2 was detected with goat anti-human ACE2 (red) antibody followed by Alexa Fluor 647 dye-conjugated goat anti-rabbit IgG. Viral N antigen (yellow) was detected with a mouse antibody against the SARS-CoV-2 N protein followed by Alexa Flour 555 dye-conjugated goat anti-mouse IgG. Nuclei were stained with DAPI (magnification: 630×.). Control: the archived normal tissues.

Upon euthanizing animals on 3, 5, 7, 9 and 21 dpi, gross examination of both lungs from all animals displayed red lesions, and severe scattered pleural adhesions could be found in SARS-CoV-2-infected rhesus macaques on 5 dpi (S2 Fig). Microscopic examination of the lung tissue revealed patchy areas of edema as mild to marked interstitial pneumonia (Fig 4A). The two animals’ necropsies on 5 dpi showed severe interstitial infiltration with diffuse alveolar damage or thickening of the alveolar. Alveoli contained a large amount of mononuclear/macrophage and neutrophil accumulation (Fig 4A, 5 dpi). Animals euthanized on 3 dpi displayed moderate interstitial infiltration, with one animal (R02) having severe infiltration in lung tissues (Fig 4A, 3 dpi). On 7dpi and 9dpi, the animals’ lung pathological symptoms did not worsen, and displayed moderate to mild alveolar inflammation (Fig 4A). However, we found that this inflammation could not be completely resolved by 21 dpi, with thickening of alveolar and local inflammatory infiltration that remained in the lung tissues (Fig 4A, 21 dpi). Meanwhile, damage to epithelial cells of tracheal tissue was found on 3–9 dpi (Fig 4B). We also observed histological changes of LNs, including tracheal and lung LN, which displayed immune activity by enlarged germinal centers in response to viral infection (Fig 4C). Analysis of these sections for the expression of viral antigens by immunohistochemistry (IHC) yielded positive results for the presence of the SARS-CoV-2 N antigen in lung, trachea and LNs of animals on 3–9 dpi (Fig 4D, 4E and 4F). To further confirm that the virus replicated in lower respiratory tract, we performed confocal microscopy analysis on the SARS-CoV-2 N antigen and Angiotensin I converting enzyme 2(ACE2) colocalization. On 3, 5, 7, 9 and 21 dpi, viral N antigen was found in alveolar pneumocytes and tracheal epithelial cells corresponded with the distribution of ACE2 receptor for SARS-CoV-2 (Fig 5). Moreover, histological analyses showed mild hyperemia and edema in CNS, liver and spleen tissues from some animals, with other organs showing normal histology (S3 Table).

### Systemic and local immune responding to SARS-CoV-2 infection displayed differentially T, B and NK cells

**Fig 6:**
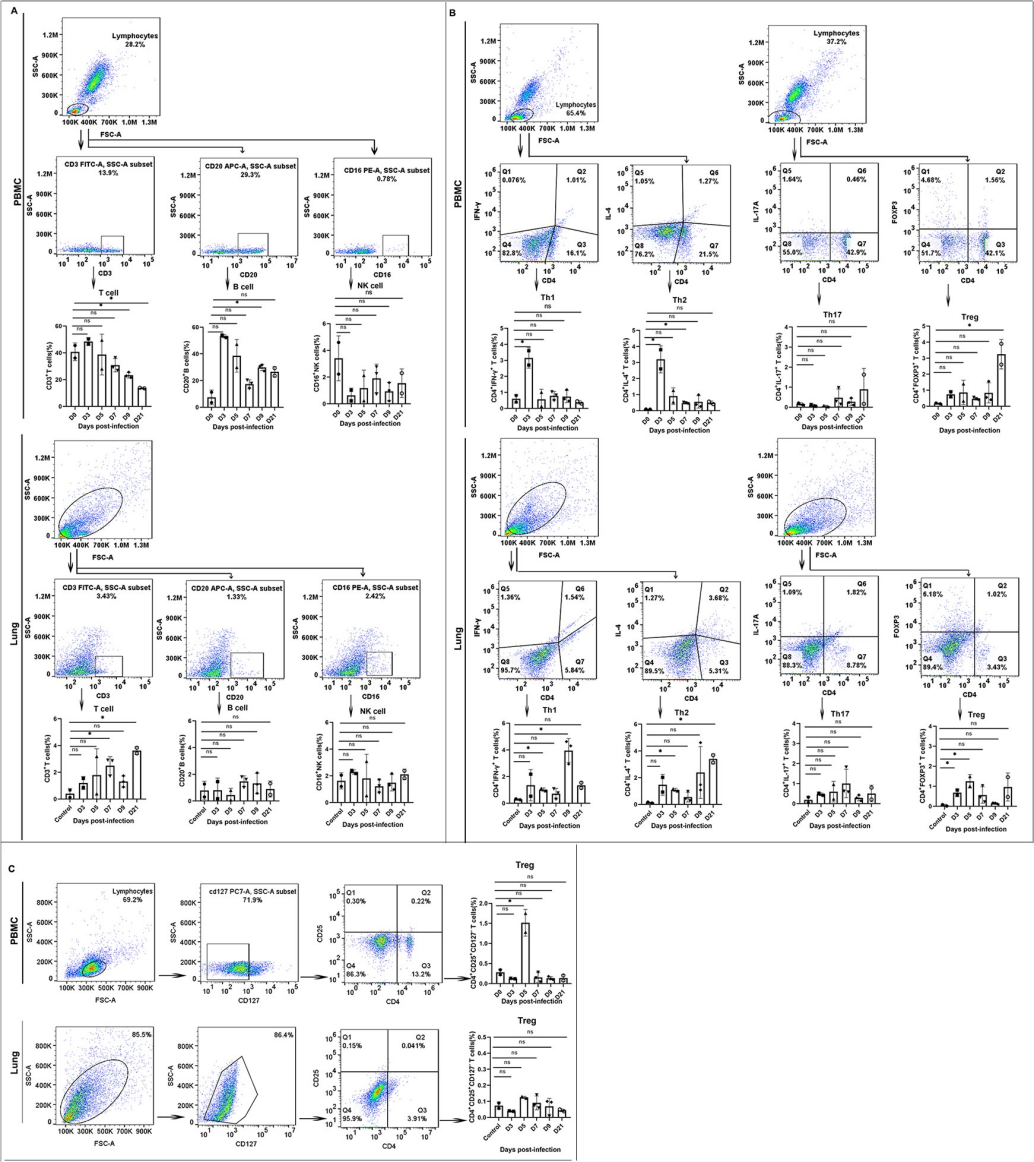
Immune analysis of SARS-CoV-2-infected rhesus macaques. (A) Lymphocyte phenotyping by T, B, and NK cells in PBMCs (upper row) and lung tissues (lower row). (B) T helper cells for IL-4, IFN-γ, IL-17 and FOXP3 expression gated in CD4+ cells from PBMCs (upper row) and lung tissues (lower row). (C)Treg cells for CD25 expression gated in CD4+CD127- cells from PBMC(upper row), and lung tissues(Lower row). Control: the archived normal lung tissues. D0: Samples from day 0(samples prior to infection)were used as controls.”*”, *p*<0.05; “ns”, *p*>0.05.

Understanding immune responses in a SARS-CoV-2 infection animal model is important for the development of vaccines and treatments for COVID-19. Based on the above pathological and pathogenic results of SARS-CoV-2 infected rhesus monkeys, we analyzed the proportion of CD3^+^T cells, CD20^+^B cells and CD16^+^NK cells in PBMC on 3–21 dpi. Several clinical studies have reported that CD3^+^T and CD20^+^B cells from severe patients were significantly lower than those from mild patients[24]. Compared with the normal cell proportion range in PBMC, we found a decrease in lymphocytes following SARS-CoV-2 infection, which is similar to findings in COVID-19 cases[8, 25]. Remarkably, the proportions of T cells and B cells increase on 3 dpi(*p*<0.05), and all decreases occur gradually as the severity of lung injury increases. In contrast, NK cells in PBMC show a decreasing trend from the initial 3 days of infection and do not recovered until 21 dpi. (Fig 6A). The proportion of T cells in lung tissues continued to increase during the infection period(*p*<0.05, 5 and 21 dpi). These data were in accordance with the findings of lymphocyte infiltration in the lung under microscopic examination.

### T cell subsets analysis

Several studies have shown that CD3^+^T cells, especially CD4^+^ T cells, are associated with the pathogenesis of severe SARS-CoV-2 infection[25, 26]. However, data describing the kinetics of the T cell response during SARS-CoV-2 infection are lacking. Our above findings suggest that the decrease in T cells could contribute to the outcome of SARS-CoV-2 infection. We monitored the T cell profile, including Th1, Th2, Th17 and Treg cells, in the CD4^+^ T cells from PBMC and lung tissue on different days post infection. The results indicate that both CD4^+^IFN-γ^+^ Th1 and CD4^+^IL-4^+^ Th2 in PBMC were increased on 3 dpi (*p*<0.05)but gradually decreased as the severity of the illness increased in the following days. Furthermore, increases in CD4^+^Foxp3^+^ Treg cells could be observed from 3 to 21 dpi (*p*<0.05, 21 dpi) with no significant change in the proportion of CD4^+^IL-17^+^ Th17 cells in PBMC (Fig 6B). Notably, CD4^+^CD25^+^CD127^-^ Treg cells also exhibited an increased trend on 5 dpi (*p*<0.05) (Fig 6C), which is when severe interstitial pneumonia occurred. The above findings suggest that the antiviral response of T cells was suppressed after 3 days post infection, which might be related to the increased Treg cell population in PBMC. Intriguingly, compared with the increase in the above Th1 and Th2 cells in PBMC during the early stage of infection, we found that the Th1 and Th2 cells in the lung tissue obviously increased during the late stage of infection(*P<0*.*05*) (9 dpi, 21dpi), contributing to the local immune response of the animals during infection and indicating that the local T cell response of cytokine secretion may be related to lung pathogenesis.

### Cytokine Profiles

**Fig 7:**
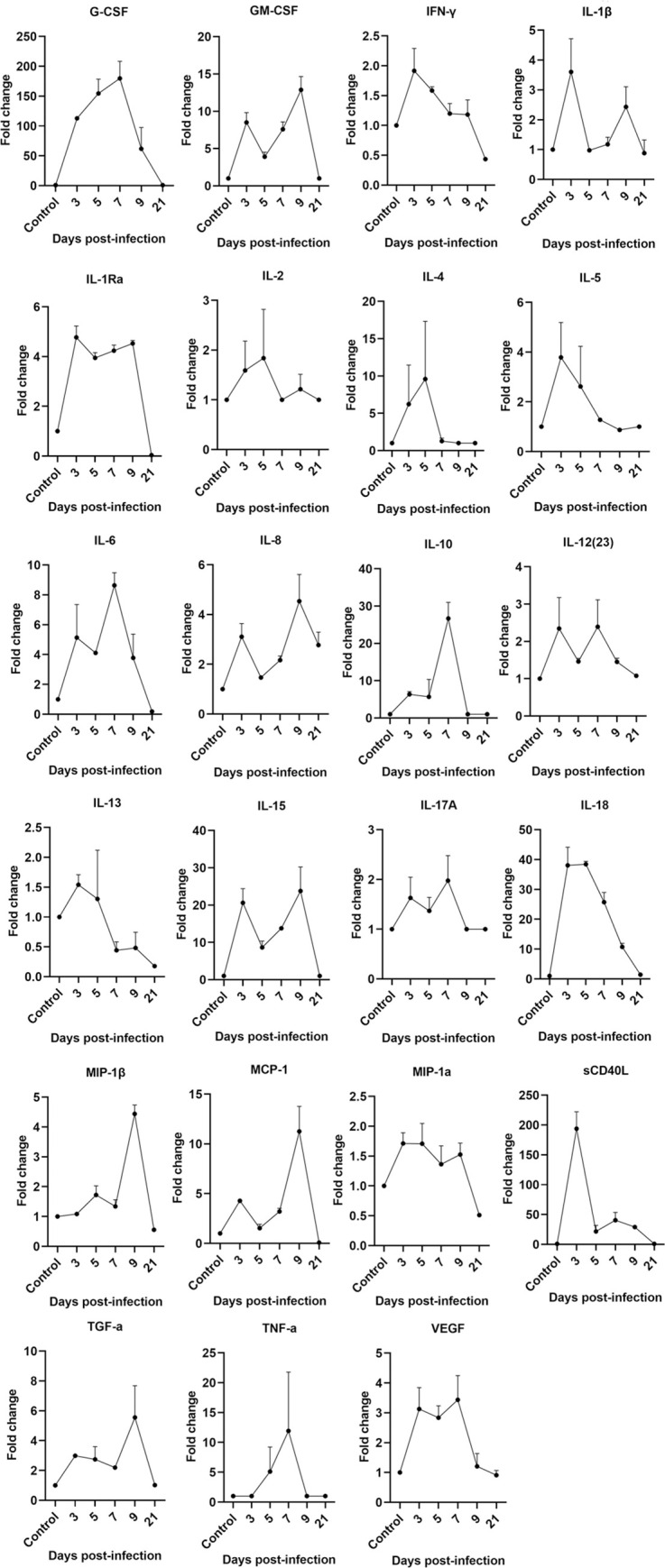
Changes of cytokines/chemokines concentrations during the SARS-COV-2 infected process. Cytokine cytometric bead arrays were performed to measure the concentrations of 23 cytokines and chemokine. The fold change of cytokines of each samples = The concentration value of each experimental well /the concentration value of the control well. The concentration below the detection line is calculated as the concentration of limit of detection. Three multiple wells are set for each sample, and the mean±SD are finally calculated. Control: the archived normal lung tissues.

Recent clinical studies have suggested that Th1/Th2 cytokines might play a key role in SARS-COV-2 infection[27]. We determined the serum cytokines at 0, 3, 5, 7, 9 and 21 dpi. IL-2, IL-4, IL-5, IL-6, IFN-γ, and TNF-αwere found to be obviously higher on 3, 5, 7, and 9 dpi and returned to the normal level on 21 dpi (S3 Fig). To further identify the inflammatory mediators involved in SARS-COV-2-induced pneumonitis in rhesus macaques, we measured the protein levels of local cytokines and chemokines secreted in the lung tissues at 0, 3, 5, 7, 9 and 21 dpi, and 23 cytokines and chemokines, including proinflammatory cytokines (IL-1βand IL-6), Th1 cytokines (IFN-γ, TNF-α, IL-2, and IL-12), Th2 cytokines (IL-4, IL-5, IL-10, and IL-13), IL-17 cytokines and chemokines (MIP-1β/CCL4, MCP-1/CCL2, MIP-1α/CCL3, and IL-8/CXCL8) etc., were observed on 3–9 dpi compared to archived normal tissues control. First, an obvious increase in the levels of IFN-γ, IL-6, IL-1β, IL-1Ra, IL-18, IL-5, IL-8 was detected in the early (3 dpi) stage of infection (Fig 7), indicating the activation of innate immune responses, such as monocytes and NK cells. The expression level of IL-17A did not significantly change after infection, which is consistent with the trend of the changes in Th17 cells. Second, there was a significant increasing trend in the Th1/ Th2 cytokines IL-2, TNF-a, IL-4, and IL-10 on 5–7 dpi during the middle stage of infection (Fig 7). We conclude that Th1 and Th2 cells obviously increase in the middle stage of infection in lung tissue, contributing to the local immune responses of animals during infection and indicating that local T cells respond in terms of cytokine secretion. Finally, a marked increase in inflammatory chemokines MIP-1β/CCL4, MCP-1/CCL2, and IL-8/CXCL8, accompanied by an increase of inflammatory cytokines IL-15, IL-12, TNF-a, GM-CSF, and G-CSF, which was detected in the lung predominantly at day 7–9, the middle and late stage of infection. Interestingly, we found some cytokines (GM-CSF, TGF-α, IL-6, IL-1β, IL-15, et al) or chemokines (MCP-1, MIP-1a, et al.) showed two waves of production enhancement.(Fig 7)The first wave of these cytokines and chemokines was activated early on 3 dpi and sharply decreased on 5–7 dpi, when the severe pneumonia was observed in lung tissues. By day 7–9, when moderately pneumonia was observed in the lung tissues, the second peak of the production of these cytokines was detected.

At the gene transcription level, the cytokine profiles of PBMC in SARS-CoV-2-infected monkeys showed an obviously increasing level on 3 dpi, particularly as the IL-6 and IL-1β expression level increased by 900- and 191-fold (S4 Fig), respectively, compared with that on 0 dpi and 3 dpi, which is also consistent with the various trends observed in the lung tissues. Therefore, IL-6 and IL-1 beta may serve as a reference for SARS-COV-2 infection. Collectively, our data show that some proinflammatory cytokines and Th1 and Th2 cytokines might play a certain role in SARS-COV-2 infection.

### Detection of the neutralizing antibody level in rhesus monkey infected with SARS-CoV-2

**Fig 8:**
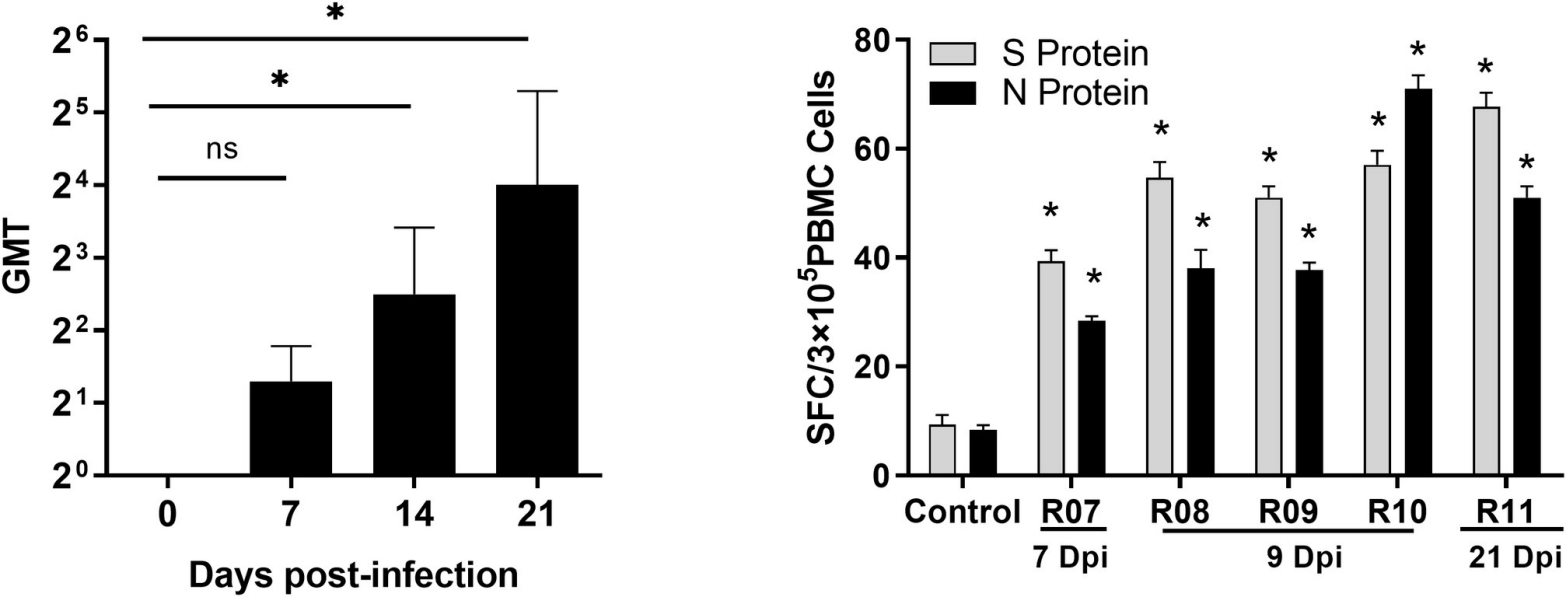
Neutralizing antibody level and ELISpot analysis of rhesus macaques infected with SARS-CoV-2. (A) Neutralizing antibodies against SARS-CoV-2 were titered on Vero cells at 100 TCID_50_ virus. (B) S and N protein-specific IFN-γ+ secretion T cells were numbered on precoated PVDF plates by an ELISpot reader. Compared with controls,”*”, *p*<0.05; “ns”, *p*>0.05.

On 21 dpi of SARS-CoV-2 infection, the geometric mean titer (GMT) of the neutralizing antibody levels was detected to be approximately 1:5.6–16 (Fig 8A), suggesting that SARS-CoV-2 infection in the rhesus monkeys elicited an adaptive immune response in the animals. Furthermore, we obtained S and N protein-specific IFN-γ+ T cells from the PBMC of the infected animals on 7, 9, and 21 dpi, reveling 30–80 SFC per 3×10^5^ PBMC (*p<*0.05) (Fig 8B).

## Discussion

The first isolate of SARS-CoV-2 was obtained from a patient with fatal pneumonia in December 2019[28]. Subsequently, COVID-19 has threatened more than 20,000,000 patients globally, with a case-fatality rate of 2.3%[3]. Multiple clusters reporting infection provided solid evidence of person-to-person transmission[14, 29]. Clinically, patients with COVID-19 present lower respiratory symptoms due to interstitial pneumonia and radiologically multifocal ground-glass opacities, even in patients with mild disease[14, 30]. The severity of the disease of COVID-19 appears to be more similar to that of severe respiratory disease caused by severe acute respiratory syndrome (SARS). However, SARS-CoV-2 differs from MERS-CoV and SARS-CoV due to its long viral shedding and asymptomatic infections[30, 31] and is becoming the main threat to public health in the future.

To interpret the process of infection and understand the systematic pathology of the disease caused by SARS-CoV-2 in humans, an effective SARS-CoV-2 infection animal model is urgently needed. To date, nonhuman primates, such as Macaca mulatta and Macaca fascicularis, have been used as animal models in studies investigating SARS-CoV-2 infection[19, 22]. Compared to data reported by other studies, we summarized different models, including monkey species, infection route and dose, host immune response, pathological changes of lung tissue (Table 1), virus shedding, viremia, and viral tissue distribution (Table 2). In our study, the nasally-infected rhesus macaques displayed similar clinical and pathological manifestation of COVID-19, especially lower respiratory tract disease proceeding from mild to marked pneumonia. In addition to the progression of the infection, we observed virus shedding predominantly via the nose and oropharynges that lasted for 27 and 14 days, respectively. We also observed two peaks of viral RNA from nasal swabs as follows: the first peak of the viral RNA should be the input of the virus, while the second peak was due to authentic viral replication, which is consistent with the other studies [19, 22]. Notably, we found that virus shedding from the stool may also contribute to virus spreading, virus shedding continued up to 27 days from 9 dpi, and the infectious virus could be isolated on 9dpi; some clinical studies have reported that the infectious SARS-CoV-2 virus was long-standing in human stool samples[32, 33]. Based on these studies, we posit that the role of fecal excretion, even the waste water in the spread of SARS-CoV-2 cannot be ignored.

**Table 1:** Host immune response and pathological observation in non-human primates of SARS-CoV-2 infection.

Monkey species/age	Inoculation Route/Dose	Clinical Symptoms	Gross pathology examinations of lung	Microscopic pathology examinations of lung	Host immune response Refs			
					**In PBMC**^**a**^	**In serum**^**a**^	**In lung**^**b**^
Rhesus macaque 8–12 months	IN; 2×10^5^TCID_50_	less activity and loss of appetite; fever	Red lesions, severe scattered pleural adhesions	Interstitial infiltration, diffused alveolar damage, thickening of alveolar, Alveoli contained mononuclear/macrophage and neutrophils accumulation	**3dpi:** CD3^+^T↑,CD20^+^B↑, CD16^+^NK↓, CD4^+^IFNγ^+^ T↑, CD4^+^IL-4^+^T↑; **5-7dpi:** CD3+T↓,CD20+B↑, CD16+NK↓,CD4+CD25+CD127-T ↑ **9-21dpi:** CD3+T↓, CD20+B↑, CD16+NK↓; CD4^+^FOXP3^+^T↑	**3dpi:**IL-2↑, IL-4↑,IL-5↑, IL-6↑, TNF-α↑, IFN-γ↑ **5-7dpi:** IL-2↑, IL-4↑, TNF-α↑, IFN-γ↑,IL-5↓,IL-6↓ **9-21dpi:IL-2(-), IL-4**↑, IL-5↓, IL-6↓, TNF-α↑, IFN-γ(-) ,	**3dpi:** G-CSF↑,GM-CSF↑,IFN-γ↑,IL-1β↑,IL-1Ra↑,IL-5↑,IL-6↑,IL-8↑,IL-10↑,IL-12↑,IL-13↑,IL-15↑,IL-18↑;MCP-1↑,sCD40L↑, TGF-α↑,VEGF↑ **5-7dpi:** G-CSF↑, GM-CSF↑,IFNγ(-),IL-1β(-),IL-Ra↑, IL-2(-), IL-4(-), IL-5(-), IL-6↑,IL-8↑, IL-10↑, IL-12↑, IL-13↓,IL-15↑,IL-17A↑, IL-18↑, MIP-1β(-),MCP-1↑, MIP-1α(-), sCD40L↑, TGF-α(-),TNF-α↑, VEGF↑, **9-21dpi:** G-CSF(↓), GM-CSF(↓-),IFNγ↓,IL-1β(↓),IL-Ra↓, IL-2(-), IL-4(-), IL-5(-), IL-6↓,IL-8↑, IL-10(-), IL-12(-), IL-13↓,IL-15(↓),IL-17A(-), IL-18(↓), MIP-1β(↓),MCP-1(↓), MIP-1α↓, sCD40L(↓), TGF-α(↓),TNF-α(-), VEGF(-)	This study
macaca fascicularis 4-5years; 15-20years	IN, IT; 10^7^TCID_50_	Nasal discharge; No significant weight loss	foci of pulmonary consolidation	Hyaline membranes, Alveolar edema, Leukocyte infiltration	No data	No data	No data	19
Rhesus macaque 4–6 years	IN, IT, oral, Oc; total 6ml of 4×10^5^ TCID_50_/ml	Irregular respiratory patterns change; piloerection; reduced appetite; hunched posture; pale appearance; cough; weight loss; fever;	Varying degrees of gross lung lesions	Interstitial pneumonia, edema fluid, fibrin, macrophages and neutrophils accumulation	No data	1dpi:IL-1ra↑,IL-6↑,IL-10↑,IL-15↑,MCP-1↑,MIP-1b↑; 3dpi: TGF-a↓;	No data	20
Rhesus macaque 6-11years	IT; 7×10^6^CCID_50_	Reduced appetite	large patchy hemorrhage; red dark foci; consolidation	Necrosis, collapse and fibrosis, extensive fibroblast proliferation, inflammatory cells infiltration	No data	No data	No data	22
Rhesus macaques 3–5 years; 15 years	IT; 10^6^ TCID_50_	Weight loss, fever, Asthenia,	patchy lesions; ground-glass opacities	Pneumonia, inflammatory cells infiltration	**2-4dpi:** CD3^+^CD4^+^ T↓; CD3+ CD8+ T↓ (in young monkeys)	No data	No data	21
Rhesus macaques macaca fascicularis, Callithrix jacchus Young/Adult/Aged	IT, Oc, IN; 1×10^6^ -4.75×10^6^ PFU	High body temperature, loss of body weight;	nodules, masses, and interstitial patterns	Pulmonary septum, the infiltration of inflammatory cells, diffuse hemorrhage, necrosis	**Increase(2-4dpi):**CD4^+^T↑, CD8^+^T ↑, CD14^+^↑ **Decrease(6dpi):** CD4^+^T↓, CD8^+^T ↓, CD14^+^ ↓	Eight cytokines(G-CSF,IL-1A,IL-8,IL-15,IL-18,MCP-1,MIP-1B, and sCD40L) were detected in most of the infected monkeys	No data	23

**Note:** IN, intranasal; IT, intratracheal; OC, ocular; PFU, plaque-forming units; TCID_50_, 50% tissues culture infectious dose; Refs, references.; ↑,increasing;↓,decreasing; (-),return to normal level

a: 0 dpi samples (samples prior to infection) as controls.

b: Archived normal tissues as controls

**Table 2:** Viral shedding and distribution in non-human primates of SARS-CoV-2 infection.

Monkey species	Viral duration/peak viral load^a^			Viremia	Virus distribution in tissues	Infectious Virus shedding	Refs
	Nasal swabs	Oropharyngeal swabs	Rectal swabs				
Rhesus macaque	1-27dpi, 7.5×10^5^	2-14dpi, 8.7×10^4^	9-27dpi, 1.9×10^5^	On 5dpi,	Lung, Trachea, Intestine, CNS, Trachea LN, Neck LN, Lung LN, Kidney, Liver, Spleen, Heart, Testical	Rectal swabs	This study
macaca fascicularis	1-18dpi, 10^3.4^–10^4.7^ TCID_50_ equivalent/ml	1-10dpi, 10^2.8^–10^3.9^ TCID_50_ equivalent/ml	On 14dpi,	No viremia	Respiratory tissues, ileum, colon, tonsil.	Throat swabs, Nasal swab,	19
Rhesus macaque	1-15dpi, about 10^4^−10^8^	1-15dpi, about 10^4^−10^8^	3-17dpi, about 10^2^−10^5^	No viremia	Respiratory tract tissues, Stomach, Ileum, Colon, Cecum, Cervical LN, Mediastinal LN, Conjunctiva, Heart, Tonsil	Nose swabs;Balf fluid; Lung;	20
Rhesus macaque	1-9dpi, about 10^4^−10^7^	1-11dpi 2.08×10^4^–3.6×10^7^	2-11dpi, about 10^5^	No viremia	Lung, Trachea and bronchus tract	Nasal swabs, Oropharyngeal swabs	22
Rhesus macaques	3-11dpi, about 10^4^−10^8^	3-11dpi, about 10^4^−10^8^	3-11dpi, about 10^4^−10^6^	No data	lung	no data	21
Macaca mulatta/ macaca fascicularis/ Callithrix jacchus	2-21dpi, ~5.0×10^11^	2-17dpi,21dpi Variously in different groups	2-14dpi,18dpi,21dpi; Variously in different groups	2-10dpi	Lung, Trachea, Bronchus, Stomach, Rectum, cerebrospinal fluid, Intestine Ln, hilar lymph node, Bladder, Spleen,et al.	No data	23

Abbreviations: LN, lymph nodes; Refs, references.

a SARS-COV-2 RNA was determined by qRT-PCR, copies/ml

The shedding pattern observed in the rhesus macaques is strikingly similar to that observed in humans[32]. In humans and rhesus macaques, the SARS-CoV-2 virus can be detected in both the upper and lower respiratory tracts. We also found viremia transiently positive on 5 dpi in this model, indicating the potential of systemic viral spreading in the organs of SARS-CoV-2 infected animals. This speculation was supported by the observation of the wide distribution of virus RNA dominant in the respiratory tract and spreading to other tissues. The expression of SARS-CoV-2 viral antigens was positive in the lung, trachea and lung lymph node, further indicating viral dissemination. Such predominant replication of SARS-CoV-2 in the respiratory tract and oropharyngeal and nasal virus shedding may support person-to-person transmission. In addition to lung and LN tissue, we found virus distributed in gastrointestinal tissue, CNS tissue, and other main tissues and organs, which is consistent with other studies[20, 22]

In this rhesus macaque model, the progression of pneumonia was recapitulated and displayed typical pathology of interstitial infiltration with diffused alveolar damage in the lung. Remarkedly, this progression revealed a moderate-severe-moderate-mild process on 3, 5, 7, and 9 dpi. Although improvement and lung tissue recovery were observed, such progression could not be completely resolved by 21 dpi. This unsatisfactory outcome of COVID-19 in the rhesus macaque model may hopefully serve as a reference for clinical treatment and vaccine evaluations.

Virus dynamics and host response are essential for formulating strategies for antiviral treatment, vaccination, and epidemiological control of COVID-19. In the study of SARS-CoV and MERS-CoV, severe disease in clinical cases or macaque models is associated with the immune response[34, 35] might be related to a higher viral load, prolonged viral shedding, lung pathologic changes and an impaired antibody response. In this case, the T cell response in SARS patients during the acute phase has been reported to be suppressed[36]. Particularly, Treg cells have a high capacity to produce cytokines that act as regulators of leukocyte trafficking in SARS-COV-2-infected patients[25]. In our rhesus macaque model, we found that a proportion of T cells, including Th1/Th2/Th17 cells, displayed a reduction during the middle and late stages of infection, while the CD4+FOXP3+T cell response was activated during this period. Our results further indicate that CD4+CD25+ CD127- Treg cells show an increasing trend on 5 dpi, confirming their activation during the middle stage of infection, which may lead to immunosuppressive effects consistent with clinical reports[25]. NK cells in PBMC showed a decreasing trend during the initial 3–5 days of infection, indicating that the innate response was limited during the early phase of infection.

Significantly, we revealed that the local immune response to SARS-CoV-2 infection differed from the above PBMC reaction. Th1, Th2 and Th17 cells were increased in the lung tissue from 3 dpi, indicating that local T cells responded via cytokine secretion and lung pathogenesis. Several studies have indicated that acute respiratory distress syndrome (ARDS) is the ultimate result of the cytokine storm in the most severe cases of SARS-COV-2 infection[37]. In this study, local cytokines/chemokines in lung, such as IFN-γ, IL-4, IL-6, IL-1β, MCP-1, and IL-8, MIP-1a, were increased by more than 2-fold within 3 days post SARS-CoV-2 infection. We hypothesize that this wave of increased expression of cytokines/chemokines might contribute to the recruitment of other inflammatory cells, such as mononuclear/macrophages, neutrophils and NK cells, when lung injury begins. As previous study reported in an mice model of SARS infection [38], we also observed a second wave of inflammatory molecules on days 7–9, which involved an increase in the cytokines IL-15, IL-17A, IL-12, and TNF-a and chemokines MIP-1β/CCL4, MCP-1/CCL2, and IL-8/CXCL8 and coincided with lung tissue injury and the infiltration of inflammatory lymphocyte cells. By 21 dpi (the late stage of infection), most chemokines and cytokines returned to the normal level, and the symptoms of pneumonia were mild as the infiltration of lymphocytes decreased. According to these data, we conclude that inflammatory mediators, including cytokines, chemokines and immune cells, may play important roles in mediating the local immune response in lung tissue.

By collecting data in our study, we provide a rhesus macaque animal model recapitulating the SARS-CoV-2 infection process by mimicking natural nasal virus inoculation. Notably, long-term viral shedding (27 dpi) in nasal swabs was observed, in accordance with the report of 24 d viral shedding in nasopharyngeal aspirates after symptom onset in human case[31]. We also observed the interstitial pneumonia in rhesus macaques, which is a typically pathological changes in humans. More importantly, we emphasized Treg cell functioning critically in COVID-19 progression[39]. We summarized, in addition to focusing on virus shedding and interstitial pneumonia, the T cell response and local cytokine/chemokine changes, which should be critical characteristics in a successful animal model of COVID-19 that could be applicated in vaccine and treatment evaluations.

### Limitations of this study

There are several limitations in our study. First, we used small/young/immature/male animals (8–12 months) to establish a SARS-CoV-2-infected rhesus macaque model. During the COVID-19 outbreak epidemic, our research team had limited availability of animals to carry out our experiments, so we chose young/male animals to perform experiments, which do not comprehensively reflect the influence of sex and age on SARS-CoV-2 infection. Second, the viral load of pharyngeal swab and nasal swab samples was reflected by viral gene copies, we did not use viral infectious titers, as only the viral load is partially reflected by viral proliferation. Finally, we observed changes only in the number of T cell subsets, and it was found that changes in the number of Treg cells were associated with severe infection; however, the specific role of Treg cells in the infection process is not clear.

## Materials and methods

### Biosafety and animal ethics

All work with infectious SARS-CoV-2 was performed with approval under Biosafety Level 3 (BSL3) and Animal Biosafety Level 3 (ABSL3) conditions by the Institutional Biosafety Committee of Institute of Medical Biology (IMB). The BSL3/ABSL3 facilities have been designed to conform to the safety requirements recommended by the China National Accreditation Service for Conformity Assessment (CNAS) and the National Health Commission (NHC) of the People’s Republic of China (PRC). The facility and laboratory safety plans have been approved for use by the NHC and CNAS. Experiments with infectious virus were performed in a certified Class IIB biosafety cabinet in BSL3. Within the ABSL3 facilities, all members of staff wore scrubs, 3M or Tyvek suits and waterproof gowns, powered air-purifying respirators, shoe covers, two sets of gloves for monkey experiments. Staff members were subject to a medical surveillance plan monitored by the IMB, and any symptoms associated with SARS-CoV-2 infection were reported and investigated by IMB when working in the BSL3/ABSL3. All animal experiments were approved by the Institutional Animal Care and Use Committee of IMB.

### Virus and cells

The viral strain SARS-CoV-2-KMS1/2020 (GenBank accession number: MT226610.1) was isolated from sputum collected from a COVID-19 patient by IMB&CAMS, and propagated and tittered on Vero cells in DMEM (Sigma, USA) supplemented with 2% (vol/vol) FCS (Gibco, Australia), 50 U/mL penicillin, and 50 μg /mL streptomycin (Gibco, Australia) at 37°C with 5% CO2. The stock virus titer was 10^6.0^TCID_50_/ml and freezing at -80°Cprepared for the all following experiments.

### Animal study and sample collection

Fourteen male rhesus macaques (age:8–12 months, weight:2–3 kg) were used in this study. Experimental infections were performed via the nasal route using 200μl of the SARS-CoV-2-KM/2020 strain (2×10^5^ TCID_50_). Animals were anesthetized by intramuscular injection with 10 mg/kg of ketamine hydrochloride. Swab samples (nasal swab, oropharyngeal swab, and rectal swab) and sera were collected at 1–11, 14, 17, 19, 21, 24, and 27 dpi. The whole blood was collected in K_2_EDTA tubes for viral RNA extraction and FACS analysis. Twelve animals were assigned randomly for scheduled necropsies on 3, 5, 7, 9, and 21 dpi, and two additional animals were monitored for survival. In the experiments containing tissue samples, we used archived normal tissues as controls. For the samples from whole blood (serum, PBMCs), samples from day 0(samples prior to infection)were used as controls.

### SARS-CoV-2 RNA detection by qRT-PCR

RNA from oropharyngeal, nasal, and rectal swab samples and whole blood were extracted using TRIzol reagent (Tiangen, China) following vortexing. Total RNA of tissue was extracted by homogenizing mechanically using iron beads. Two SARS-CoV-2 RNA standards were generated for transcription by ORF1a regions transcribed in vitro. ORF1ab-F: 5’-CCCTGTGGGTTTTACACTTA-3’; ORF1ab-R: 5’-ACGATTGTGCATCAGCTG-3’; ORF1ab-P: 5’-CCGTCTGCGGTATGTGGAAAGGTTATGG-3’. For quantitation of viral RNA, a standard curve was generated using 10-fold dilutions of RNA standard, and qRT-PCR was performed using a TaqMan Gene Expression Kit (Takara, China), primers (1 μM) and a probe (250 nM). Assay sensitivity was 50 copies/100μl.

### Histopathologic analyses

For histopathologic examination, various tissue samples from necropsied rhesus macaques were fixed in 10% neutral buffered formalin, paraffin-embedded, sectioned at 4 μm, and stained with H&E.

### Immunohistochemistry (IHC)

**Immunohistochemistry (**IHC) staining was performed on sections of paraffin-embedded tissue from SARS-CoV-2-infected animals that were deparaffinized in xylene, rehydrated in a graded series of ethanol and rinsed with double-distilled water. The sections were incubated with rabbit anti-N antigen of SARS-CoV-2 for 1 hour after heat-induced epitope retrieval. Antibody labeling was visualized by the development of DAB. Digital images were captured and evaluated by a histological section scanner (Aperio Digital Pathology, Leica).

### Immunofluorescence (IF)

The paraffin-embedded tissue sections were dewaxing, repairing antigen, and then the tissue sections were blocking for 2 hours in 10% FBS at RT. The slides were then incubated with anti-ACE2 antibody (Abcams, USA) for two hours at 1:500 dilution, RT. After washing three times with PBST, the tissue slides were permeabilized with 0.1% Triton-X100 for 15 min and labeled with antiviral N protein (Sino Biological, China) at 1:500 dilution for two hours at RT. Finally, the ACE2 and SARS-CoV-2 N protein antigens were visualized by Alexa Fluor 647-conjugated goat anti-Rabbit IgG and Alexa Fluor 555-conjugated goat anti-mouse IgG at 1:1000 dilution for one hour, respectively. The cell nuclei were stained with 4’,6-diamidino-2-phenylindole (DAPI, Abcam, USA). The images were captured by a Leica TCS SP8 laser confocal microscope.

### Immunophenotyping

The number of lymphocytes, including T, B and NK cells, was determined using a NHP TBNK cocktail (BD Biosciences, USA). The T help cell phenotuping was quantified using nonhuman primate CD4 (PerCP-conjugate), CD4(PE-conjugate), CD4(BV421-conjugate), IL-4(PE-Cyanine7-conjugate), IFN-γ(PE-conjugate), IL-17(eFlour660 -conjugate), FOXP3(Alexa Fluor 488-conjugate), CD25(PE conjugate), CD127(PE-Cyanine7-conjugate). All of these antibodies were purchased from BD Biosciences, USA. For staining, 0.1 ml of EDTA-anticoagulated whole blood samples were incubated for 15 min at RT in the presence of a TBNK cocktail of antibodies against CD3 (FITC conjugate), CD20 (APC conjugate), CD16 (PE conjugate), or CD4 (Percp/PE conjugate). Red blood cells were lysed using BD Pharm Lyse, after which they were washed twice in media and fixed with 0.125 ml of 2% paraformaldehyde for 15 min. After an additional wash, the cells were permeabilized using BD permeabilization/fixation reagent. The cells were stained for 15 min with anti-IL-4, anti-IFN-γ, anti-IL-17 and anti-FOXP3 while the permeabilizer was present. The cells were then washed twice in media and resuspended in 0.125 ml of 2% paraformaldehyde until they were run on a Beckman flow cytometer. Flow cytometry data were analyzed using FlowJo version 10.6.1.

### Serum Th1/Th2 cytokine analysis

Concentrations of Th1 and Th2 cytokines in serum were measured using the Non-Human Primate Th1/Th2 cytometric bead array (CBA) Kit (BD Biosciences, USA) according to the manufacturer’s instructions. The limits of detection for each cytokines were 3.6pg/ml(IL-2), 0.9pg/ml(IL-4), 0.3pg/ml(IL-5), 0.1pg/ml(IL-6), 0.4pg/ml(TNF), 3.3pg/ml(IFN-γ).

### Cytokine cytometric bead array

The concentrations of 23 cytokines and chemokines of lung tissues were measured using the Bio-Plex non-human primate cytokine magnetic bead panel (Millipore, USA) according to the manufacturer’s instructions. Then, run the plate on Luminex 200. The sensitivities of cytometric bead array for each cytokines were 2.1pg/ml(G-CSF), 1.8pg/ml(GM-CSF),1.6pg/ml(IFN-γ), 1.2 pg/ml(IL-1β), 2.4pg/ml(IL-1ra), 2.1pg/ml(IL-2), 3.1pg/ml(IL-4), 1.5pg/ml(IL-5), 1.6pg/ml(IL-6), 1.1pg/ml(IL-8), 6.4pg/ml(IL-10), 1.5pg/ml(IL-5), 5.8pg/ml(IL-13), 0.5pg/ml(IL-15), 1.3pg/ml(IL-17A), 3.1pg/ml(MCP-1), 1.6pg/ml(MIP-1β), 4.9pg/ml(MIP-1α), 2.1pg/ml(sCD40L), 1.1pg/ml(TGF-α), 1.6pg/ml(TNF-α), 13.6pg/ml (VEGF), 6.1pg/ml(IL-18).

### PCR array analysis of PBMCs

RNA extracted from PBMCs was run for cytokine and chemokine expression of rhesus macaques by PCR array analysis using the primers listed below (Table 3). 2^-△△CT^(Livak) method was used to calculate the change of genetic level, we used beta-actin as the reference gene.

**Table 3:** Primers for different kinds of cytokine and chemokine genes.

Primer	Sequence (5’-3’)
IL-8-F	ttttgccaaggagtgctaaaga
IL-8-R	aaccctctgcacccagttttc
IFN-α-F	gcctcgccctttgctttact
IFN-α-R	ctgtgggtctcagggagatca
IFN-β-F	atgaccaacaagtgtctcctcc
IFN-β-R	ggaatccaagcaagttgtagctc
IFN-r-F	tcggtaactgacttgaatgtcca
IFN-r-R	tcgcttccctgttttagctgc
IL-1β-F	atgatggcttattacagtggcaa
IL-1β-R	gtcggagattcgtagctgga
IL-2-F	tacaagaacccaaactgactcg
IL-2-R	acatgaaggtagtctcactgcc
IL-4-F	ccaactgcttccccctctg
IL-4-R	tctgttacggtcaactcggtg
IL-6-F	actcacctcttcagaacgaattg
IL-6-R	ccatctttggaaggttcaggttg
IL-17A-F	tcccacgaaatccaggatgc
IL-17A-R	ggatgttcaggttgaccatcac
IL-22-F	gcttgacaagtccaacttcca
IL-22-R	gctcactcatactgactccgt
TNF-a-F	cctctctctaatcagccctctg
TNF-a-R	gaggacctgggagtagatgag

### Neutralization assay

Serum samples were serially diluted two-fold in 96-well microplates, and 50 μl of SARS-CoV-2 containing 100 TCID_50_ was added to 50 μl of each serum dilution and incubated at 37°C for 2 hours. Supernatants were then transferred to microtiter plates containing confluent Vero cell monolayers. After incubation for 4 d, titers were calculated as the reciprocal of the serum dilution.

### ELISpot

SARS-CoV-2-specific cellular immune responses were assessed by interferon-γ (IFN-γ) ELISpot assays using peptides against the S and N proteins. Ninety-six-well precoated plates (R&D, USA) were incubated with 2 μg/ml of each peptide and 3 × 10^5^ monkey PBMCs in triplicate in 100 μl reaction mixture volumes. Following an 18-hour incubation at 37°C, the plates were washed and incubated with anti-IFN-γ (BD Biosciences, USA) for 2 hours at RT followed by washing with PBST and incubated for 2 hours with a 1:500 dilution of streptavidin-alkaline phosphatase. Following development and air drying, the plate was read by an ELISpot reader (CTL, USA). The numbers of SFCs per 3×10^5^ cells were calculated.

### Statistical analyses

Analysis of virologic and immunologic data was performed using GraphPad Prism v8.0 (GraphPad Software, CA, USA). The Student’s t test is used for statistical analysis by SPSS.23.

## Supporting information

S1 Fig Sequence comparison of S (A), E (B), and nucleocapsid N (C) genes of SARS-CoV-2 isolated from nasal swab samples on 3, 9 and 14 dpi.
(TIF)

S2 Fig Gross pathology examination of lung from rhesus macaques at 5 dpi (B). Compared with a normal lung (A)
(TIF)

S3 Fig CBA analysis of Th1/Th2 cells for IL-2, IL-4, IL-5, IL-6 and TNF in serum samples. The fold change of cytokines of each samples = The concentration value of each experimental well /the concentration value of the control well.
(TIF)

S4 Fig PCR array for Cytokine and chemokine profiles in PBMC. 2^-△△CT^(Livak) method was used to calculate the change of genetic level, we used beta-actin as the reference gene, and then value of Day 0 as the baseline and control value. The fold change of cytokine levels of D3, D5, D7, D9, D21 was compared with the Day0. For performing relative quantitative analysis, the CT value of target gene and internal reference gene value of the experimental samples and control samples were normalized: △△CT = △CT_(Targeted sample)_-△CT_(D0),_ Then, the expression ratio is calculated: 2^-△△CT^ = 2^-[△△CT = △CT(Targeted sample)-△CT(D0)]^
(TIF)

S1 Table Scoring criteria for evaluating clinical signs. (DOCX)

S2 Table The infectious viral titer in rectal swabs. The supernatant from swabs were used for TCID50 assay on vero cells.
(DOCX)

S3 Table Histological analyses of other organs of rhesus macaques inoculated with SARS-CoV-2. (DOCX)
