# Supplementary figures and images for: Virulence and pathogenesis of SARS-CoV-2 infection in rhesus macaques: A nonhuman primate model of COVID-19 progression
Source: PLoS Pathog. 2020 Nov 12;16(11):e1008949. doi: 10.1371/journal.ppat.1008949 (PMC7660522; doi:10.1371/journal.ppat.1008949)

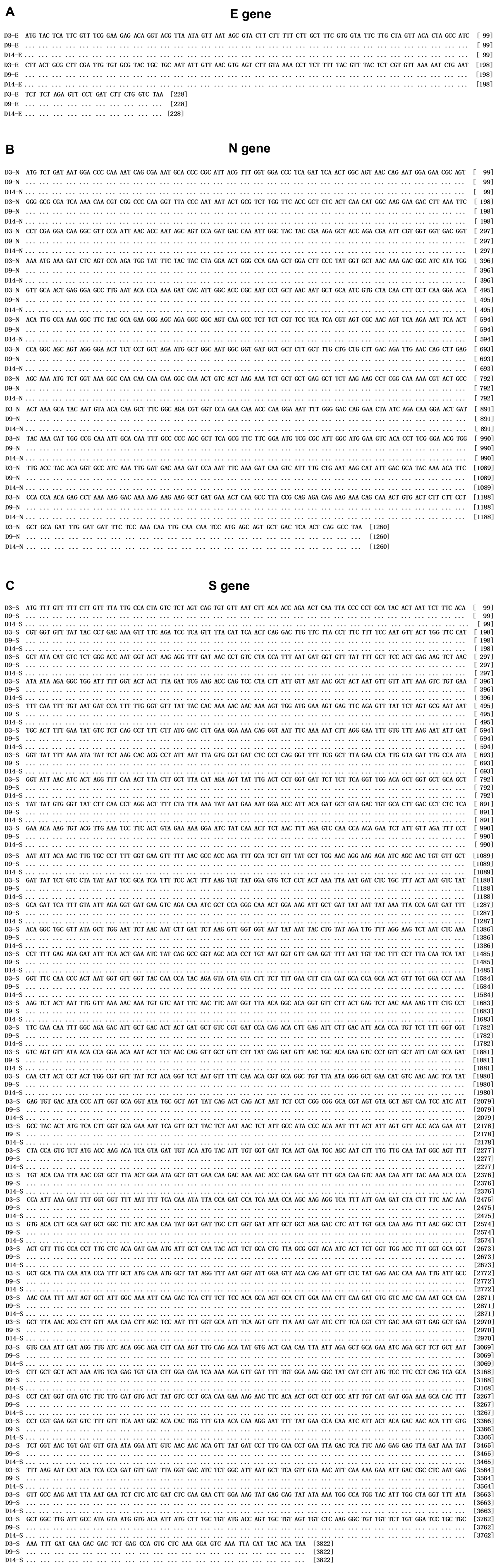

Supplement: S1 Fig — Sequence comparison of S (A), E (B), and nucleocapsid N (C) genes of SARS-CoV-2 isolated from nasal swab samples on 3, 9 and 14 dpi. (TIF) [file ppat.1008949.s001.tif]

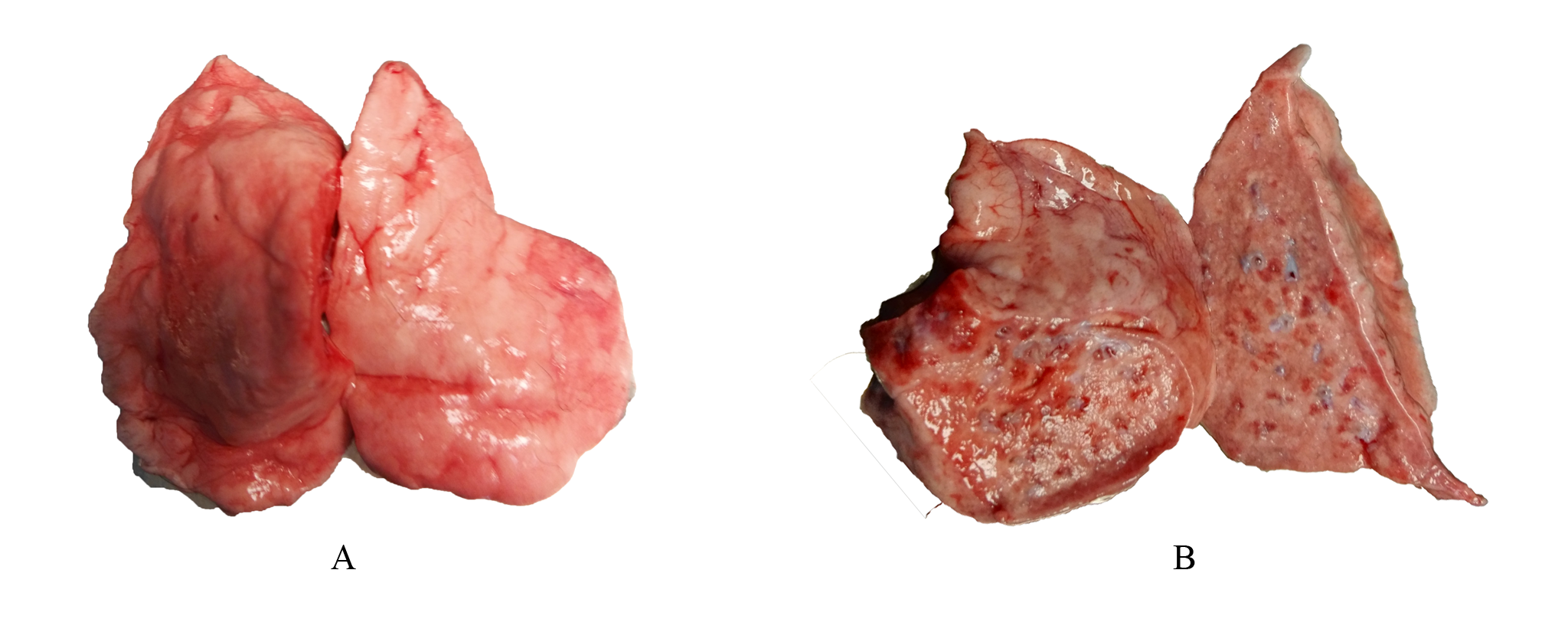

Supplement: S2 Fig — Gross pathology examination of lung from rhesus macaques at 5 dpi (B). Compared with a normal lung (A) (TIF) [file ppat.1008949.s002.tif]

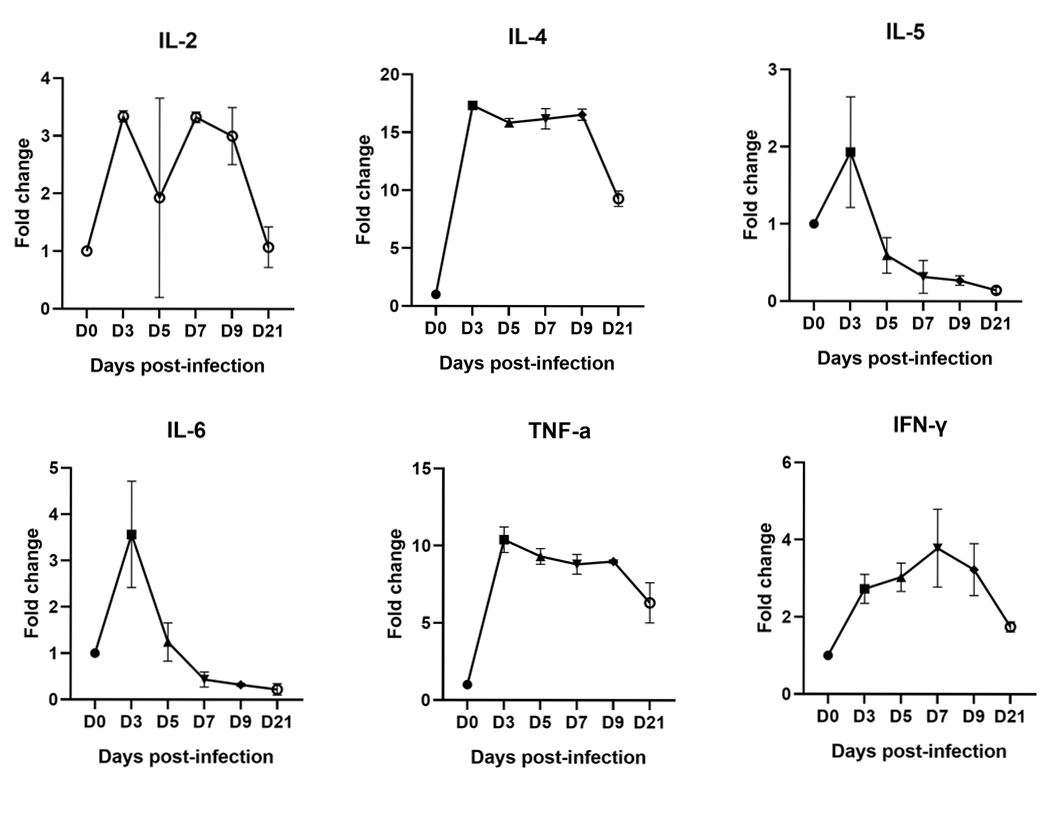

Supplement: S3 Fig — The fold change of cytokines of each samples = The concentration value of each experimental well /the concentration value of the control well. (TIF) [file ppat.1008949.s003.tif]

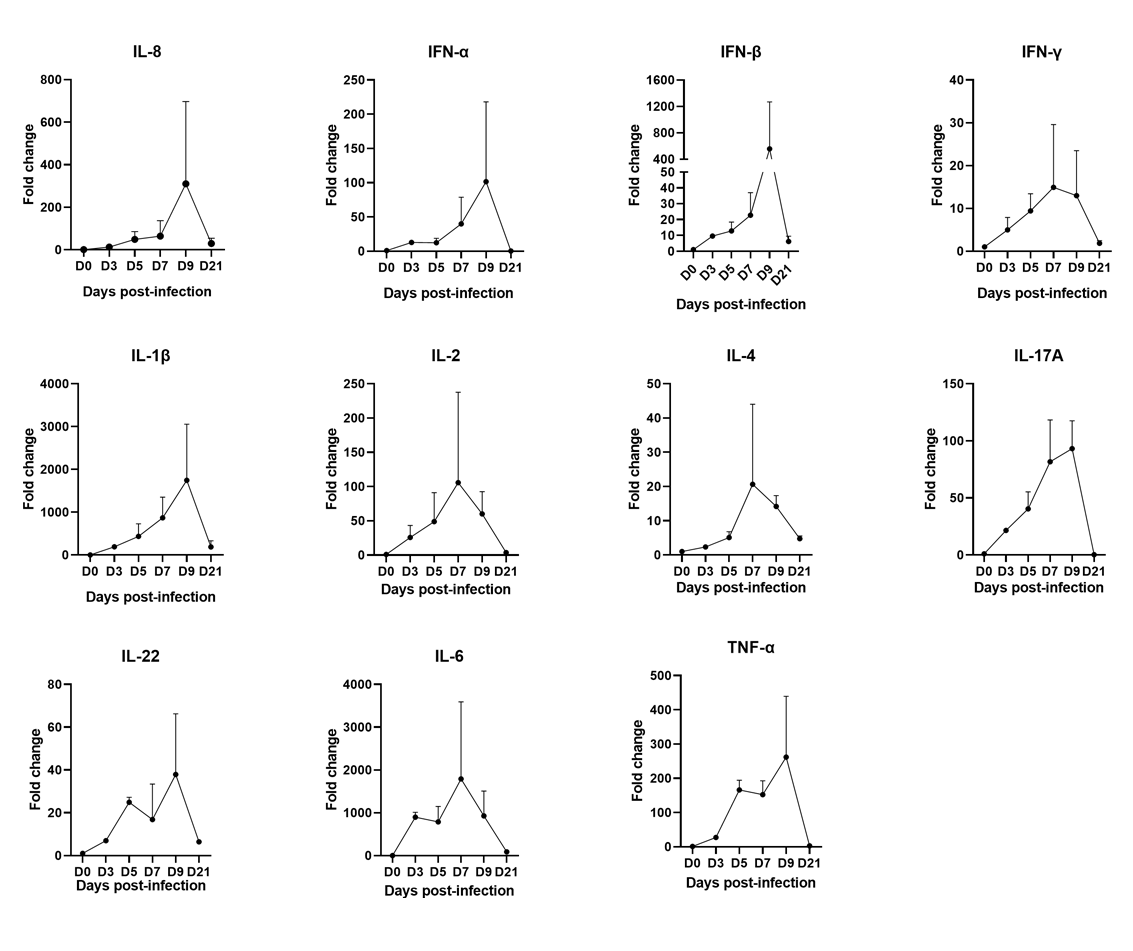

Supplement: S4 Fig — 2-△△CT(Livak) method was used to calculate the change of genetic level, we used beta-actin as the reference gene, and then value of Day 0 as the baseline and control value. The fold change of cytokine levels of D3, D5, D7, D9, D21 was compared with the Day0. For performing relative quantitative analysis, the CT value of target gene and internal reference gene value of the experimental samples and control samples were normalized: △△CT = △CT(Targeted sample)-△CT(D0), Then, the expression ratio is calculated: 2-△△CT = 2-[△△CT = △CT(Targeted sample)-△CT(D0)] (TIF) [file ppat.1008949.s004.tif]
