# Supplementary material for: Virulence and pathogenesis of SARS-CoV-2 infection in rhesus macaques: A nonhuman primate model of COVID-19 progression
Source: PLoS Pathog. 2020 Nov 12;16(11):e1008949. doi: 10.1371/journal.ppat.1008949 (PMC7660522; doi:10.1371/journal.ppat.1008949)
Supplement: S1 Table — (DOCX) [file ppat.1008949.s005.docx]

Table S1. Scoring criteria for evaluating clinical symptoms

|  | **Score** |
| --- | --- |
| **General Appearance** |  |
| Normal and alert, moving without prompting | 0 |
| Slow/ quiet, hunched, but alert, interested, moving without prompting | 5 |
| Quieter, hunched , but alert ,moving needs a lot of prompting | 10 |
| Loss of interest, almost impossible to prompt to move, dull expression, falling asleep while watched, little or no response to human presence | 15 |
| **Food consumption** |  |
| Normal appetite | 0 |
| Loss of appetite | 2 |
| Anorexia | 5 |

Note； This scoring criteria table is cited from the reported animal evaluated criteria in other studies [18]
