# Supplementary material for: Virulence and pathogenesis of SARS-CoV-2 infection in rhesus macaques: A nonhuman primate model of COVID-19 progression
Source: PLoS Pathog. 2020 Nov 12;16(11):e1008949. doi: 10.1371/journal.ppat.1008949 (PMC7660522; doi:10.1371/journal.ppat.1008949)
Supplement: S2 Table — The supernatant from swabs were used for TCID50 assay on vero cells. (DOCX) [file ppat.1008949.s006.docx]

| **Table S2:** The viral infectious titers in rectal swabs from SARS-COV-2 infected rhesus macaque | | | | | |
| --- | --- | --- | --- | --- | --- |
| **Macaque ID** | 3 dpi | 9 dpi | 14 dpi | 19 dpi | 24 dpi |
| R11 | **-** | 100 | **-** | **-** | **-** |
| R12 | **-** | **-** | **-** | **-** | **-** |

**Note:** The unit of the viral titer is TCID_50_/ml;”**-**” represents that no infectious virus was detected in rectal swabs.
