# Supplementary material for: Virulence and pathogenesis of SARS-CoV-2 infection in rhesus macaques: A nonhuman primate model of COVID-19 progression
Source: PLoS Pathog. 2020 Nov 12;16(11):e1008949. doi: 10.1371/journal.ppat.1008949 (PMC7660522; doi:10.1371/journal.ppat.1008949)
Supplement: S3 Table — (DOCX) [file ppat.1008949.s007.docx]

Table S3. Histological analyses other organs rhesus macaques inoculated with SARS-CoV-2

| **ID** | **Heart** | **Liver** | **Spleen** | **I**[**ntestine**](javascript:;) | **Kidney** | **Medulla oblongata** | [**Thalamus**](javascript:;) | **Pons** | **Midbrain** | **Cerebellum** | [**Thoracic**](javascript:;)  [**cord**](javascript:;) | **Lumbar myeloid** | [**Cervical**](javascript:;)  [**cord**](javascript:;) |
| --- | --- | --- | --- | --- | --- | --- | --- | --- | --- | --- | --- | --- | --- |
| **R01** | **+** | **+** | **+** | **-** | **-** | **+** | **+** | **-** | **-** | **-** | **-** | **+** | **-** |
| **R02** | **-** | **+** | **+** | **-** | **-** | **+** | **+** | **-** | **+** | **-** | **-** | **+** | **-** |
| **R03** | **+** | **+** | **-** | **-** | **-** | **-** | **+** | **-** | **-** | **-** | **-** | **-** | **-** |
| **R04** | **-** | **-** | **+** | **-** | **-** | **-** | **-** | **-** | **-** | **-** | **-** | **+** | **-** |
| **R05** | **-** | **+** | **+** | **-** | **-** | **-** | **-** | **-** | **-** | **-** | **-** | **-** | **-** |
| **R06** | **-** | **+** | **+** | **-** | **-** | **-** | **-** | **-** | **-** | **-** | **-** | **-** | **-** |
| **R07** | **+** | **+** | **+** | **-** | **-** | **-** | **-** | **-** | **-** | **-** | **-** | **-** | **-** |
| **R08** | **+** | **+** | **+** | **-** | **+** | **-** | **+** | **-** | **-** | **-** | **-** | **-** | **+** |
| **R09** | **+** | **+** | **+** | **-** | **-** | **-** | **-** | **-** | **-** | **-** | **-** | **-** | **-** |
| **R10** | **-** | **+** | **+** | **-** | **-** | **-** | **-** | **-** | **-** | **-** | **-** | **-** | **-** |
| **R11** | **-** | **+** | **-** | **-** | **-** | **-** | **+** | **+** | **-** | **-** | **-** | **-** | **-** |
| **R12** | **-** | **+** | **-** | **-** | **-** | **-** | **-** | **-** | **-** | **-** | **-** | **-** | **-** |

Note: The “+”represent the pathologic change of the organ. The number of “+” represent the degree of the pathologic changes, the “+”represent the slight pathologic change, the “++”represent the moderate pathological change, “+++” represent the severe pathological change. The”-” represent no pathological change.
